# P04.82. Yoga for breast cancer: a systematic review of randomized controlled trials

**DOI:** 10.1186/1472-6882-12-S1-P352

**Published:** 2012-06-12

**Authors:** H Cramer, S Lange, P Klose, A Paul, G Dobos

**Affiliations:** 1 University of Duisburg, Complementary and Integrative Medicine, Essen, Germany

## Purpose

To systematically review the effectiveness of yoga in patients with breast cancer.

## Methods

MEDLINE, PsychInfo, EMBASE, CAMBASE, and the Cochrane Library were screened through September 2011. Randomized controlled trials (RCTs) comparing yoga to controls were analyzed. Risk of bias was assessed using the Cochrane risk of bias tool. For each outcome, standardized mean differences (SMD) and 95% confidence intervals (CI) were calculated, if it at least 2 studies assessing this outcome were available. As a measure of heterogeneity, I<sup>2</sup> was calculated.

## Results

11 RCTs and 655 subjects were included. 7 RCTs compared yoga to wait-list control groups, 3 RCTs compared yoga to supportive therapy and 1 RCT compared a combination of physiotherapy and yoga to physiotherapy alone.

Yoga compared to control showed significantly greater improvements in global health-related quality of life (HRQoL) (SMD=0.62, [95% CI 0.04, 1.21] p=0.04, I^2^=79%), as well as in functional (SMD=0.30 [95% CI 0.03, 0.57], p=0.03, I^2^=0%), social (SMD=0.29 [95%CI 0.08, 0.50], p=0.006, I^2^=0%), and spiritual HRQoL (SMD=0.41 [95% CI 0.08, 0.74], p=0.01, I^2^=0%). Greater improvements were also found in anxiety (SMD=-1.51 [95% CI -2.47, -0.55], p=0.002, I^2^=94%), depression (SMD=-1.83 [95% CI -3.13, -0.53], p=0.006, I^2^=95%), perceived stress (SMD=-2.13 [95% CI -3.48, -0.78], p=0.002, I^2^=91%), psychological distress (SMD= -1.10 [95% CI -1.77, -0.43], p=0.001, I^2^=85%), and fatigue (SMD=-0.33 [95% CI -0.65 to -0.01], p=0.04, I^2^=49%). No significant group differences were found in physical, emotional and mental HRQoL, breast cancer specific concerns and sleep disturbances.

## Conclusion

There is encouraging evidence that yoga has beneficial effects on HRQoL, psychological health and fatigue in breast cancer patients. Due to methodological and statistical heterogeneity, larger studies with rigorous trial design and reporting are necessary to underpin these results.

